# Risk adjustment is crucial in comparing outcomes of various surgical modalities in patients with ileal perforation

**DOI:** 10.1186/1754-9493-2-31

**Published:** 2008-11-24

**Authors:** Ravindra Singh Mohil, Tanveer Singh, Satyavrat Arya, Dinesh Bhatnagar

**Affiliations:** 1 Department of Surgery, V.M. Medical College and Safdarjang Hospital, New Delhi 110029, India

## Abstract

**Background:**

Using crude mortality and morbidity rates for comparing outcomes can be misleading. The aim of the present study was to compare the outcome of various surgical modalities without and with risk adjustment using Physiologic and Operative Severity Scoring for the enUmeration of Mortality and morbidity (POSSUM) score in cases of ileal perforations.

**Methods:**

Prospective study on 125 patients of ileal perforations. Resection anastamosis (Group I) was done in 38 patients, primary repair (Group II) in 42 patients and 45 patients had an ileostomy (Group III). The disease severity was assessed in all patients using POSSUM score. The odds of death without and with risk adjustment using POSSUM mortality score were calculated for all groups

**Results:**

Seventeen patients (14%) patients died and 99 (79%) developed postoperative complications. Using crude mortality rates Group I appeared to be the best treatment option with only 2 (5%) deaths followed by Group II with 5 (12%) deaths where as Group III had the worst outcome with 10 deaths (22%). However, Group III (ileostomy) patients had higher mean POSSUM mortality and morbidity score (55.55%, 91.33%) than Group I (28%, 75.26%) and Group II (27%, 73.59%). Taking Group I as the reference (odds ratio, OR1) odds of death were greatest in Group III (OR 5.14, p = 0.043) followed by Group II (OR 2.43, p = 0.306). With risk adjustment using POSSUM mortality score the odds of death decreased in Group III (OR 1.16 p = 0.875). For the whole group, there was a significant association between the POSSUM score and postoperative complications and deaths. Mean POSSUM mortality and morbidity score of those who died (63.40 vs.33.68, p = 0.001) and developed complications (66.32 vs.84.20, p = 0.001) was significantly higher. For every percent increase in severity score the risk of postoperative complications and death increased by 1.10 (p = 0.001) and1.06 (p = 0.001) respectively.

**Conclusion:**

Despite ileostomy patients having highest crude mortality and complication rates, after risk adjustment it was equally safe. Severity of the disease rather than the surgical option had a significant impact on the outcome in patients with ileal perforations.

## Background

Peritonitis arising from ileal perforations continues to be a common surgical emergency. It is caused by a variety of etiological factors and despite tremendous advances in antimicrobial agents and supportive care; it continues to have high morbidity and mortality. In India, unlike the western countries where tuberculosis and typhoid are almost unknown, these continue to be endemic and are the most commonly seen causes of intestinal perforation [1]. Typhoid fever is an acute systemic infection caused by the bacterium *Salmonella enterica *serovar Typhi. *Salmonella enterica *serovars Paratyphi A, B, and C cause the clinically similar condition, paratyphoid fever. Typhoid and paratyphoid fevers are collectively referred to as enteric fevers. According to global estimates, there are 16 million new cases of typhoid fever around the world and approximately 600,000 of these die every year. The most lethal complication of enteric fever are intestinal hemorrhage and ileal perforation, both arising from necrosis of Peyer's patches in the terminal ileum [2].

Tuberculosis can involve any part of the gastrointestinal tract and is the sixth most frequent site of extrapulmonary involvement. Both the incidence and severity of abdominal tuberculosis are expected to increase with increasing incidence of HIV infection. Tuberculosis bacteria reach the gastrointestinal tract via haematogenous spread, ingestion of infected sputum, or direct spread from infected contiguous lymph nodes and fallopian tubes. The gross pathology is characterized by transverse ulcers, fibrosis, thickening and stricturing of the bowel wall, enlarged and matted mesenteric lymph nodes, omental thickening, and peritoneal tubercles.

Paustian and Bockus [3] classified tubercular enteritis into three types according to the gross appearance i.e, ulcerative, hypertrophic & ulcero-hypertrophic. The most common site of involvement is the terminal ileum and cecum. Viable bacilli reach the ileocecal region protected by their fatty capsule. This area is most commonly infected because it has abundant lymphoid tissue with physiological stagnation and increased rate of absorption [4,5]. The involvement of jejunum and appendix uncommon, while involvement of ascending colon, rectum, esophagus and stomach rare. The lesions in small intestine are usually ulcerative while in cecum they are hypertrophic [4]. The reported incidence of perforation varies from 0–11% [4,6]. In spite of antitubercular chemotherapy perforations might occur. Though perforations are usually single, multiple perforations have also been reported [6,7]. The tubercular perforation can occur either through the tubercular ulcer or it can occur proximal to a stricture, in which case there will be an associated proximal dilatation of the bowel.

A variety of factors determine the outcome in patients with ileal perforation undergoing emergency laparotomy. Age, delay between onset of complaints and presentation in emergency, general condition of the patient, any previous treatment and last but not the least the type of surgical option exercised [8]. The surgery for ileal perforations should be simple yet effective [9]. A number of studies have been undertaken the world over to find the best surgical treatment for ileal perforation [10-17], each one claiming one modality to be more efficacious than the other. Each modality has its own drawbacks manifested in the form of leak rates, duration of hospital stay and increased morbidity and mortality. While comparing the outcomes using overall morbidity and mortality for each of the treatment arm may appear simple it can be misleading [14-18]. Meaningful comparisons between various modalities can only be made when some risk adjustment is done based on the severity of the disease [19].

Various scoring systems have been devised for risk stratification and comparison based on the perceived risk and severity of the disease e.g. APACHE II [20], POSSUM [21-23] (Physiologic and Operative Severity Scoring for the enUmeration of Mortality and morbidity) and its variant P-POSSUM [24,25] have been widely used since it was first introduced by Copeland et a [15] in 1991 for its simplicity and added advantage of considering the operative findings while calculating the risk severity. The POSSUM score was developed as an attempt to quantify the quality of surgical care and to allow comparison between different surgeons, units, hospitals and regions. The ideal risk assessment tool should be quick and easy to use, widely applicable, include elective and emergency work and accurately predict outcome. The initial researchers examined 62 factors. As in many similar areas of mathematical predication, multi-variate analysis was able to identify the most powerful predictors and reduced these to just 12 physiological and 6 operative parameters. Other factors no doubt do predict outcome but duplicate these 18 and offer no additive predictive power. Each of the 18 factors was divided into two, three or four levels and computer analysis calculated that a weighting of 1, 2, 4 or 8 approximated well to the relative predictive power, much simplifying the calculation. (Table 1)

**Table 1:** Physiologic and Operative Severity Scoring for the enUmeration of Mortality and morbidity (POSSUM) score

Physiological score				
Score →	1	2	4	8

Age	≤ 60	61–70	≥ 71	

Cardiac signs Chest radiograph	No failure Normal	Cardiac drugs or steroids	Peripheral edema, anticoag treatment Borderline cardiomegaly,	↑JVP, Cardiomegaly

Respiratory history	No dyspnoea	Dyspnea on exertion	Limiting dyspnea (One flight)	Dyspnoea at rest (>30/mt)

Chest radiograph	Normal	Mild COAD	Moderate COAD	Any other change

B.P(Systolic) mm Hg	110–130	131–170 100–109	≥ 171 90–99	≤ 89

Pulse	50–80	81–100 40–49	101–120	≥ 121 ≤ 39

GCS	15	12–14	9–11	≤ 8

Hb	13–16	11.5–12.9 16.1–17	10–11.4 17.1–18	≤ 9.9 ≥ 18.1

WBC(×10^3^/cu mm)	4–10	10.1–20 3.1–4.0	≥ 20.1 ≤ 3.0	

Bl. Urea	≤ 7.5	7.6–10	10.1–15.0	≥ 15.1

Na+	≥ 136	131–135	126–130	≤ 125

K+	3.5–5.0	3.2–3.4 5.1–5.3	2.9–3.1 5.4–5.9	≤ 2.8 ≥ 6.0

ECG	Normal		Atrial fibrillation (rate 60–90)	Any other abnormal rhythm or ≥ 5 ectopics/min, Q waves or ST/T wave changes

Operative Severity Score				

Score →	1	2	4	8

Operative severity	Minor	Moderate	Major	Major+

Multiple procedure	1		2	>2

Total blood Loss	≤ 100	101 – 500	501 – 999	≥ 1000

Peritoneal soiling	None	Minor Serous fluid	Local Pus	Free bowel contents, pus or blood

Malignancy	None	Primary only	Nodal Mets	Distant Mets

Mode of surgery	Elective		Emergency resusc of > 2 hrs possible, Op <24 hrs after admission	Emergency surgery, 2 hrs of resusc not possible

P – POSSUM (P stands for Portsmouth, in England) is a modified form of POSSUM that uses the same 18 parameters (12 physiological and 6 operative). Only the numerical constants in the equations are modified so as to give a better predictive power [24].

Both the scores have been applied in various surgical groups in the last decade, both in elective and emergency surgeries, for risk adjustments for the purpose of audit and comparing results in similar group of patients [26].

The aim of the present study was to prospectively study the outcome of various surgical modalities i.e. primary repair/wedge excision, resection anastamosis and ileostomy in cases of ileal perforation. The outcomes based on different techniques were then compared without and with risk adjustment based on POSSUM score. The impact of disease severity on postoperative complications and deaths was also studied.

## Methods

A total of 132 patients requiring laparotomy for suspected ileal perforation were studied over a period of eighteen months in a single unit which included seven surgeons over various point of time. Two patients died before they could be operated (due to septic shock), two patients refused surgery and three patients had other causes for peritonitis. 125 patients who intra-operatively showed no cause other than ileal perforation were finally included in the study. Only patients older than 12 years were included. All patients had their physiological scores recorded on admission. The operating surgeon based on operative findings calculated operative score.

The operative procedure was decided at the time of surgery by the operating surgeon after keeping in mind the general condition and operative findings of the patient. Based on the operative procedure patients were divided in three groups. Resection anastamosis (Group I) was done in two layers. Inner layer with vicryl 3-0 suture and the outer seromuscular layer with non absorbable 3-0 silk. The mesenteric defect was closed with 3-0 silk. The resected segment thus removed was sent for histopathological examination. Primary repair (Group II) was also done in two layers using absorbable 3-0 vicryl sutures and non absorbable 3-0 silk. A biopsy was taken from the edge of perforation before closure. Loop ileostomy (Group III) was done when primary repair, wedge resection or resection anastamosis was not feasible. The loop was brought out sufficiently before everting it so that the formal stoma formed was well above the skin level leading to less skin excoriation. In case where resection was done and this was not possible the distal end was closed and the proximal end was brought out as end ileostomy. The patients were followed for a minimum of five weeks and any mortality and morbidity within a period of 30 days was noted as per the definitions of POSSUM score defined by Copeland et al [21] (Table 2). The data was entered into *Microsoft excel*^® ^for analysis. Predicted morbidity and mortality scores were calculated based on physiologic and operative scores using POSSUM & P-POSSUM equations as under:

**Table 2:** Complications recorded as per the POSSUM scoring system

1. Hemorrhage Wound, Deep, Other
2. Infection – Chest, Wound, Urinary tract, Deep, Septicemia, PUO, Other
3. Wound dehiscence- Superficial, Deep
4. Anastamotic leak
5. Thrombosis- Deep vein thrombosis, Pulmonary embolus,
Cerebrovascular accident, Myocardial infarct
6. Renal failure
7. Respiratory failure
8. Cardiac failure
9. Hypotension
10. Any other complication

**a) **POSSUM Equation for predicting **morbidity :**

log_n _R/(1-R) = -5.91 + (0.16 × physiological score) + (.19 × operative severity score)

**b) **POSSUM Equation for predicted **mortality :**

log_n _R/(1-R) = -7.04 + (.13 × physiological score) + (.16 × operative severity score)

**c) **P-POSSUM equation for predicted **mortality: **log_n_R/(1-R) = -9.065 + (0.1692 × physiological score) + (0.1550 × operative severity score).

The actual number of patients having any morbidity and mortality were recorded and compared with expected. Observed: Expected (O: E) ratio was then calculated. A score less than unity indicate overprediction and greater than one indicating underprediction, with an ideal ratio being one.

Number of patients expected to undergo any morbidity or mortality was calculated by two methods. a) *The linear analysis *method was used to calculate mortality using P-POSSUM score. The mean predicted risk for patients in each group was calculated and multiplied by the number of patients in the group to give the predicted number of patients. The predicted morbidity and mortality rates were then compared with observed rates. b) *The exponential analysis *was used to calculate morbidity and mortality using POSSUM score. In this method used by Copeland et al [17] a cut off risk of death is considered in each stage of calculation. All patients who have predicted risk of death above the cut off level are grouped together. Therefore if the cut off level being considered is 70 percent and above, the number of predicted deaths above a cut off fall below the number calculated for a higher cut off, a second calculation should begin again from the lower cut off.

The exponential method has been criticized for not being a standard statistical technique and it is difficult to give a risk score to an individual patient by this method. P- POSSUM on the other hand uses linear regression method of analysis, which is a standard statistical technique described Hosmer and Lemeshow [21]. Wijesinghe et al [27] compared POSSUM and P-POSSUM in predicting death following vascular surgery, and demonstrated good agreement between the observed and predicted number of deaths providing the correct analysis was performed i.e. exponential for POSSUM and linear for P-POSSUM. A similar outcome was observed by us in patients undergoing emergency surgery [26].

Statistical analysis was done using STATA software version 9. One way analysis of variance (ANOVA) was used to see any significant difference in means of POSSUM mortality and morbidity scores values in different surgical groups. The outcome variable is dichotomous (i.e. death or survival) and the aim of the study was to see association of various surgical modalities with outcome, the logistic regression was used. The association of different surgical methods with mortality and morbidity without and with adjusting disease severity using POSSUM score at 95% confidence interval (CI) was seen. There is a paucity of literature to define the reference group for comparing various surgical modalities in patients with ileal perforation. Therefore Group I (primary closure) with lowest mortality was taken as reference group (OR 1) arbitrarily for logistic regression analysis. A P value <0.05 was considered statistically significant.

## Results

A total of 85 male and 40 female patients were included in the study. The operative severity score was severe in all cases as per the original definitions of Copeland et al [21]. Resection and anastamosis was done in 38 patients (Group I), 42 patients had primary repair (Group II) and in 45 patients an ileostomy was made (Group III). Overall seventeen patients (13%) died within 30 days of surgery, and 99 (77%) patients developed significant complications. The distribution of mortality and morbidity with mean POSSUM mortality and morbidity score in the three groups is shown in table 3. Group III (ileostomy) patients had significantly higher mean POSSUM mortality score (55.55%) than Group I (28%) and Group II (27%). The POSSUM score was significantly different between group I & III and group II & III (p = < 0.001). For the whole group those who died had significantly higher mean POSSUM mortality score 63.40 vs. 33.68 (p = 0.001). A similar trend was seen for POSSUM morbidity score (table 3).

**Table 3:** Comparison of Mortality and Morbidity among groups

Type of Surgery	Number of patients	Mortality number (%)	Mean POSSUM score (mortality)*	Morbidity number (%)	Mean POSSUM score (morbidity)**
Group I (Resection and anastamosis)	38	2 (5.26)	28.49+/- 17.39	28 (73.68)	75.26 +/-14.02

Group II (Primary repair)	42	5 (11.9)	26.9 +/- 16.28	32 (76.19)	73.59 +/-15.69

Group III (Ileostomy)	45	10 (22.22)	55.55 +/-22.73	39 (86.66)	91.33 +/- 8.98

Total	125	17 (14)		99 (79.2)	

*, ** Using post hoc analysis Group 1 vs. Group 3 and Group 2 vs. Group 3 were significantly different

Pain abdomen was the most common symptom present in 115 (92%) of patients followed by fever in 56 (45%) patients. In patients with tubercular perforations history of fever was erratic and only seven patients had a history of pulmonary tuberculosis (past or active). One patient was on treatment for Koch's abdomen at the time of presentation. The other common complaints at presentation are enumerated in table 4.

**Table 4:** Frequency of symptoms and root causes of ileal perforation in 125 patients included in this study

**Symptoms**	**No. of patients (%)**	**Etiology**	No. of patients (%)
Pain abdomen	115 (92)	Typhoid	99 (79)

Obstipation	60 (48)	Tuberculosis	19 (15)

Fever	56 (45)	Trauma	4 (3)

Vomiting	19 (15)	Non specific	4 (3)

Loose stools	7 (5)	Abortion	2 (1.6)

The various causes of ileal perforations encountered are enumerated in table 4. In four cases the clinical and histopathological picture was inconclusive and in two patients histopatholgy showed ischemia of the bowel. There were 20 cases where serum Widal test was significant (1/60). The incidence of gas under diaphragm was found in 45 patients (36%).

A total of 99 (79%) patients developed some form of complications. Various complications encountered are shown in table 5. Besides these, two patients went into coagulation failure, five patients had impaired renal function and two had severe hepatic dysfunction. Peristomal skin excoriation, exclusive to group III was found in 12 patients out of 45 (26%). Bed sore formation was seen in 4 patients. One patient belonging to group III developed anterior abdominal wall cellulites requiring release incision. The mean duration of hospital stay was greater in Group III as compared to patients in Group I & II (19 vs. 9 days). Eleven patients underwent re-exploration for leakage in Group I and II while one patient was re-explored for proximal ileal perforation in Group III.

**Table 5:** Incidence of complications in 125 patients with ileal perforations

Complication	No. Of Patients (%)
Wound infection	52 (42)
Chest Infection	28 (22)
Wound dehiscence	25 (20)
Urinary Tract Infection	19 (15)
Anastamotic leak	12 (9)
Respiratory failure	10 (8)
Pyrexia of Unknown oOOOrigin	7 (5)
Hypotension	5 (4)
Renal failure	5 (4)
Cardiac failure	2 (1.6)

The odds of death without and with risk adjustment using POSSUM mortality score is shown in table 6. Using crude mortality rates and taking Group I as the reference, odds of death were greatest in Group III (OR 5.14, p = 0.043) followed by Group II (OR 2.43, p = 0.306). With risk adjustment the odds of death (OR 1.16) decreased in Group III although it didn't reach statistical significance (p = 0.875). There was a significant association between POSSUM mortality score and mortality. For the whole group every percent increase in severity score the risk of death increased by 1.06 (p = 0.001).

**Table 6:** Logistic regression for odds of death and complications without and with risk adjustment using POSSUM mortality and morbidity score

Group	Number of patients (%)	Odds ratio without risk adjustment (95% CI)	P value	Odds ratio with risk adjustment (95% CI)	P value
Possum mortality score				1.06 (1.03 1.09)	0.001

Group II	42 (33.6)	2.43 (0.44 13.35)	0.306	3.4 (0.51 23.60)	0.202

Group III	45 (36)	5.14 (1.05 25.16)	0.043	1.16 (0.17 7.60)	0.875

Possum morbidity score				1.10 (1.05 1.15)	0.001

Group II	42	1.14 (0.41 3.14)	0.796	1.46 (0.44 4.8)	0.53

Group III	45	2.32 (0.14 0.75)	0.141	0.41 (0.09 1.83)	0.24

A similar association was seen between various complications observed and POSSUM morbidity scores (table 6). Without risk adjustment odds of morbidity in group II (1.14) and group III (2.32) changed to 1.46 (Group II) and 0.41 (Group III) respectively with risk adjustment, though statistically not significant. For the whole group every percent increase in morbidity score the risk of complications increased by 1.10 (p = 0.001).

## Discussion

In tropical countries the incidence of ileal perforation in the emergency outnumbers any other etiological factor of peritonitis [1]. Treatment for enteric perforation should be simple yet effective [9]. This holds true not only for enteric perforation but also for all ileal perforations whatever be the etiology. The search for a single, simple and effective procedure has been elusive. Studies to find the best option for a given set of conditions do not show a significant degree of consistency, each study declared one modality to be more efficacious than the other [10,11,16,28-30].

Primary repair has been performed in various centers and numerous studies have been undertaken from time to time to prove its mettle [1,8,11,28,30-32]. Bitar and Tarpley [9] in their review have advised the same for most cases where they describe it as "doing as much as necessary but as little as possible", the intention being a swift effective operation designed to halt the contamination and remove the existing collection. Trimming the margin by 3–4 mm has also been advocated. Primary repair has been done both as single or two layers [8,33,35].

Unsatisfactory results with universal primary repair led to introduction of short-circuiting procedures such as ileo-transverse colostomy [35-37]. This was done when the bowel was friable and sutures do not hold well. Mortality with this bypass procedure was not very different from that achieved by simple closure so with passage time, this and other bypass procedures have not found universal favour [9,31,38,39].

Resection anastamosis has been strongly advocated by many authors especially in the presence of multiple ulcers or perforations, large perforation, gangrene or unhealthy gut, hemorrhage and perforation associated with stricture [10,29,32,38-42].

Initially ileostomy was advised as a primary procedure in cases where an ileo – transverse colostomy was being done [8,9]. Later it was also advised in patients with post operative fistula and as a primary procedure to avoid the morbidity due to fecal fistula. [13,41,43-45]Ileostomy has not found universal acceptance because of the problem related to its care, the perceived risk of higher mortality and a second surgery for closure. It decreases but does not completely eliminate the risk of leak [46]. We observed anastamotic leak in 11 patients, (6 Group I and 5 Group II) with mean POSSUM morbidity and mortality score of 84 and 40 respectively. Re-perforation was found in one of our patients belonging to ileostomy group. All patients with leaks required re-exploration, creation of ileostomy and none of them died [31,38,39]. Thus after years of debate and trials there appear to be no consensus regarding the best possible surgical treatment for ileal perforations [8,19,30-43].

Rahman et al [47] have reported that the kind of surgical procedure does not appear to reduce the mortality associated with enteric perforation. Mortality according to the authors is related to toxemia, septic shock and multiple organ failure and therefore uncontrollable factors make the evaluation of any surgical procedure for this condition difficult [34-37].

Unfortunately most of the studies have used crude mortality rates to compare outcomes after various types of surgeries without making any risk adjustment for disease severity. This has lead to a perceived benefit or disadvantage of one treatment arm over the other. In a recent study, Atamanalp et al [46] analysed their twenty six years experience of managing enteric perforation. Of the 82 patients studied, primary repair was done in 39%, 22% underwent either wedge resection/resection with anastamosis and 39% had an ileostomy. In their study ileostomy group had the highest mortality (7/9) followed by primary closure (2/7) with no mortality in wedge resection/resection anastamosis group. They therefore concluded that wedge resection/resection anastamosis should be the preferred operation as it had no mortality. The authors have not compared the disease severity in the various subgroups but it appears from the comparative table that majority of patients who had ileostomy presented late with moderate to severe peritoneal contamination.

In the present study too, when crude mortality rates were used for comparing outcomes, Group III (ileostomy) appeared to have the worst outcome (Table 3). The mean duration of hospital stay of 19 days was also higher in this group as compared to 9 days in the other two groups. This apparent worse outcome changed when a risk adjustment was made based on the POSSUM severity score. A similar trend was seen for morbidity where with risk adjustment the odds of morbidity decreased in ileostomy group. For the whole group irrespective of treatment arm it was observed that each percentage rise in severity score significantly increased the OR of death by 1.06. Mean POSSUM score of those who died was also significantly higher. Group III (ileostomy) patients had significantly higher mean POSSUM score (55.55%) than Group I (28%) and Group II (27%). Therefore using crude mortality and morbidity rates without considering disease severity for comparing outcomes in these groups of patients can be misleading.

The operating surgeon has to take multiple factors into consideration before choosing the type of surgical procedure. Probably no single procedure can be universally applicable to all patients with ileal perforations. Every procedure has its own advantages and disadvantages. Although it may not be possible to take any cutoff POSSUM score to choose between various treatments options we observed that primary closure is safer in patients with single perforation, healthy bowel and minimal contamination and low POSSUM score (mean 28% in present study). Resection anastamosis may be done in a similar group of patients but with multiple perforations or a severely diseased segment and healthy bowel.

Ileostomy is a safer option in patients with high POSSUM mortality scores (mean 55% in present study) and the surgeon is not sure about the integrity of the closure. Although a surgeon can identify high-risk patients even by his "gut feeling", in emergency patients he tends to underestimate the risk [48]. The POSSUM score being simple, objective may help to make a decision. To avoid morbidity related to leaks patients with high scores, adverse local findings and being operated by an inexperienced surgeon may benefit from an ileostomy. With practice the score can be calculated at the time of laparototmy within two to three minutes and has already been validated in patients undergoing emergency laparotomy in our setup [26]. The score is a good tool for audit in any surgical unit to monitor the quality of care and compare outcomes based on disease severity rather than the surgical technique used.

## Competing interests

The authors declare that there was no financial conflict of interest for all authors which includes any employment, consultancies, stock ownership, honoraria, paid expert testimony, patent applications/registrations, and grants or other funding.

## Authors' contributions

I also verify that the authors have made substantial contributions to the above study by one or more of the following:

RSM: Conception and design of the study, analysis and interpretation of data. TS: Acquisition of data, drafting the article SVA: important intellectual content, statistical analysis and interpretation of data DB: final approval of the version to be submitted. All authors have read and approved the final manuscript and agreed to the contents. I also verify that the manuscript, including tables has not been previously published and that the manuscript is not under consideration elsewhere.
